# Supervised toothbrushing programmes and at home brushing behaviour: a rapid review of evidence

**DOI:** 10.1038/s41432-026-01218-y

**Published:** 2026-04-10

**Authors:** George Kitsaras, Sarab El-Yousfi, Kara A. Gray-Burrows, Peter Day, Zoe Marshman

**Affiliations:** 1 Division of Dentistry, Faculty of Biology, Medicine and Health, The University of Manchester, Manchester, UK; 2 School of Clinical Dentistry, University of Sheffield, Sheffield, UK; 3 School of Dentistry, Faculty of Medicine and Health, University of Leeds, Leeds, UK; 4 Community Dental Service, Bradford District Care NHS Foundation Trust, Bradford, UK

**Keywords:** Oral hygiene, Dental public health

## Abstract

**Abstract:**

Supervised toothbrushing programmes (STPs) are widely implemented to improve children’s oral health, yet it remains unclear which behaviour change techniques (BCTs) they use and whether these support children’s toothbrushing at home. This rapid review addresses this evidence gap to inform programme design and evaluation.

**Aim(s):**

To conduct a rapid review of behaviour change techniques (BCTs) used in supervised toothbrushing programmes (STP1qs) and assess whether they support children’s at-home toothbrushing.

**Methods:**

A rapid review (Feb–Mar, updated Jul 2025) across PubMed, MEDLINE, Scopus and PsycINFO. Inclusion criteria focused on primary studies on children ≤12 years, STPs with any link to home brushing. Behaviour change techniques (BCTs), the active ingredients in changing behaviours, identified in included studies were code according Behaviour Change Taxonomy (BCTv1). BCTs were also mapped to COM-B mode of behaviour change and the Theoretical Domains Framework. There was no formal quality appraisal.

**Results:**

From 187 records, 153 were screened, 16 full texts assessed, and 4 studies included (randomised controlled trials, mixed-methods, single-case). A range of BCTs was identified: instruction/demonstration, behavioural practice, prompts/cues, social support, environmental restructuring; incentives/rewards appeared in some trials. Only one study explicitly used the COM-B. Evidence for effects on home brushing was limited and largely parent-reported (e.g., greater enthusiasm, longer brushing, transfer of songs/charts), with no robust quantitative measures of home routines.

**Conclusion(s):**

Based on this rapid review, a number of STPs reliably embedded BCTs strengthening children’s capability and opportunity to brush with the possibility of a “spill over” effect to home brushing behaviour. However, evidence that STB is associated with sustained at-home brushing remains weak and poorly theorised. Future evaluations should pre-specify mechanisms, measure home brushing with validated tools, and test which BCT combinations most effectively bridge school-to-home behaviour.

Key points

- STPs show indications of influencing at-home toothbrushing, but current evidence is weak, largely qualitative, and not supported by robust behavioural measurement.
- Embedding behavioural science systematically into STP design and evaluation is essential to maximise impact both in school and at home.
- More research is needed to explore, quantify and pinpoint a link between in school and at home brushing behaviour and showcase specific mechanisms of action.

## Introduction

In England, 26.9% of 5-year-old children surveyed in 2024 experienced dental caries (enamel/dentine)^1^. Significant disparities exist with children living in the most deprived areas being twice as likely to have dental caries as those in the least deprived^2^. Dental caries in children can result in short- and long-term negative implications including pain, eating^1^, sleeping difficulties^2^, changes in behaviour (e.g., irritability), adverse development on speech and language^3^, and loss of school days with an associated impact on school performance^1,4,5^. If left untreated, dental caries can progress and lead to extractions in hospital^6^ with the NHS in England spending £81 m on tooth extractions in young children per annum^1,2,6,7^.

Toothbrushing with fluoride toothpaste twice a day has been identified as a key behaviour for prevention of dental caries^8^. Supervised toothbrushing programmes (STPs) are typically delivered in early years settings, such as nurseries and schools, with children encouraged to brush their teeth with fluoride toothpaste under the supervision of trained staff. STPs have been shown to reduce both the prevalence of dental caries and inequalities, while also being cost-effective, particularly when targeted to areas of deprivation^8^. STPs have a long history of implementation in educational settings across the world with varying degree of success^8^. In England, oral health promotion is part of the Department for Education’s Early Years Foundation Stage statutory guidance, which states that early years settings must promote good health (‘including the oral health’) of the children they look after. Recently, STPs have gained momentum through policy changes at the national level. Across the UK, national STPs are already in place in Wales and Scotland, with evidence demonstrating their positive impact. In England, a national STP is being implemented across local authorities targeted to children living in the 20% most deprived areas.

Behavioural science is a key component of STPs since toothbrushing behaviours are not just about knowing how to brush, but also about wanting to do it consistently and correctly. Behavioural science helps bridge this gap between knowledge and action^9,10^. By understanding what behavioural determinants hinder or facilitate behaviour using frameworks such as the Theoretical Domains Framework (TDF)^11^, we can identify and utilise evidence-based behaviour change techniques (BCTs) that specifically address these determinants.

Generally, STPs are designed to complement home toothbrushing behaviour and to improve children’s toothbrushing behaviours more generally so they can use these skills at-home beyond the confinement of STP settings like nurseries and schools. Despite the benefits stemming from STPs, there is still an important evidence gap regarding a link between school and at home toothbrushing behaviour for children. Previous reviews in this area^12,13^ have highlighted barriers and facilitators around STPs however, and despite the emerging utilisation of behavioural sciences across oral health promotion, little is known of the specific behaviour change techniques utilised within STPs. Moreover, there is no specific review of the link between STPs and home brushing behaviour and if there are specific BCTs that can help support the transition from supervised toothbrushing in school to at-home behaviour changes.

## Aim

To conduct a rapid review of behaviour change techniques employed in supervised toothbrushing programmes with a focus on ways toothbrushing behaviours are promoted in early years settings and the home environment.

## Objectives

To identify the behaviour change techniques used within STP

To identify ways home toothbrushing is supported via the STP

## Methods

A rapid review of the available literature on supervised toothbrushing programmes for young children in school and nursery settings was conducted (between February and March and updated in July 2025). Appendix C provides a copy of a completed PRISMA checklist. Rapid reviews are often used to inform policy and use systematic methods to synthesise knowledge and provide evidence in a shorter timeframe through simplifying the review process^14^. This streamlined approach produces fewer comprehensive results than systematic reviews however it has been argued that there no significant differences in key findings^15^.

### Search strategy

The search strategy was developed using a PICO framework to ensure a structured and comprehensive approach to identifying relevant studies. The population of interest was children aged 12 years or younger, reflecting age groups commonly targeted by STPs. Interventions included any form of in-school toothbrushing delivered within early years or primary education settings including nurseries, kindergartens and schools. As this review aimed to describe the content and behavioural components of STPs rather than compare alternatives, no comparator was required. Eligible outcomes encompassed changes in toothbrushing behaviours, oral hygiene practices, acceptability of the intervention, and reported behavioural determinants or techniques underpinning programme delivery.

To operationalise the PICO structure, the search combined Medical Subject Headings (MeSH) for databases that support controlled vocabulary (e.g., PubMed) with free-text keywords relating to child populations, supervised toothbrushing, oral health promotion, and behaviour change. Boolean operators (AND/OR) were used to refine and combine terms systematically, ensuring both sensitivity and specificity. Detailed search strings are provided in Appendix A. Four electronic databases—PubMed, MEDLINE, Scopus and PsycINFO—were searched to capture studies from dentistry, public health, behavioural science, and multidisciplinary domains.

### Study selection

Studies were eligible for inclusion if they involved children aged 12 years or younger, evaluated an STP delivered in an educational setting, and reported any explicit or implicit link to home toothbrushing behaviours. Only primary research studies (including randomised controlled trials, cohort studies, case–control studies, and observational designs) and those available in full text and published in English were included. Studies were excluded if they focused on adults or children older than 12 years, did not involve supervised toothbrushing components, or were secondary research such as systematic reviews or commentaries.

Initially, a small sample of titles and abstracts was screened by two reviewers to ensure quality of screening and to resolve any issues. Then, two reviewers(GK and SE-Y) independently screened all titles and abstracts for eligibility. Full texts of potentially relevant articles were subsequently obtained and assessed against the inclusion criteria, with disagreements resolved through discussion. Data related to study characteristics for all included studies were extracted and summarised. Specific behaviour change techniques (BCTs) included in each study were identified (found in text, highlighted and listed per study with specific examples where possible/available). Then each listed BCT was mapped individually against the BCTv1 taxonomy from two reviewers (GK and KG-B) who are experts in behavioural science and psychologists working in dentistry^15^. BCTs are the active ingredients in creating and sustaining change in behavioural interventions. Where studies explicitly stated the BCTs used, this was recorded verbatim, otherwise the intervention description was coded by the researchers using the BCTv1 taxonomy^15^.

Following categorisation under BCTv1, identified BCTs were mapped against the COM-B model of behaviour change^16^, followed by an additional mapping exercise against the TDF^11^. The COM-B model explains that for any behaviour to occur, three components must be present: Capability (the knowledge and skills to perform it), Opportunity (external factors that make it possible), and Motivation (the internal processes that drive it). It is a framework used to design behaviour change interventions by identifying which of these components need to be strengthened or modified. The TDF is an integrative framework that synthesises key constructs from multiple behaviour change theories into a set of domains (such as knowledge, beliefs, social influences, and environmental context) to understand the determinants of behaviour. It is often used in implementation science to identify barriers and facilitators to behaviour change and to guide the design of interventions. The COM-B model provides a high-level view of what drives behaviour—capability, opportunity, and motivation—while the TDF breaks these components down into more detailed domains, offering a finer-grained understanding of specific influences. Used together, COM-B identifies where change is needed, and the TDF helps pinpoint what to target within those areas to design effective interventions.

Due to the nature of this rapid review and heterogeneity of selected studies no quality assessment was conducted.

### Ethics declarations

No ethical approval was sought for this work due to its nature.

## Results

### Overview of study selection and characteristics of included studies

The search yielded 187 records. After removal of duplicates, the remaining 153 titles and abstracts were screened for eligibility, with 16 eligible records identified and read in full text. Four studies were included. Appendix B summarises the flow diagram of study selection. Across these studies, work was conducted in early-years or primary-school settings and spanned Ireland, England, Scotland, and Thailand. Designs ranged from large examiner-blind, parallel-group school-based randomised controlled trials, a mixed-methods service evaluation, and a single-case experimental pre–post study. Table 1 summarises key characteristics of included studies.

**Table 1:** Supervised toothbrushing programmes: overview of included studies.

Study	Country	Setting	Design	Population (age)	Sample size	Intervention	Comparator	Duration/Date	Outcomes
Anishchuk and Waldron (2024)	Ireland	Pre-schools (17 schools; 19 classes)	Single-case experimental (pre–post)	Pre-school children; parents; pre‑school teachers	331 children; 227 parents (completed pre/post); 16 dental hygienists	Dental hygienist–supported daily toothbrushing led by teachers for 3 months; storybook, song, parent leaflets; teacher training; standards based on Childsmile	None (within-group pre–post)	3 months;	Parent/teacher knowledge & reported brushing routines
Petersen et al. (2015)	Thailand	Schools (15 schools)	Examiner‑blind parallel‑group school‑based RCT	Pre‑school children (approx. 4–6 y at baseline)	3,706 children (1,940 intervention; 1,766 control)	Enhanced OHP + closely supervised post‑lunch toothbrushing; teacher workshops; supply of 1450 ppm F + 1.5% arginine toothpaste; home supplies	Usual practice (unstructured brushing; ≤1000 ppm F toothpaste)	24 months; published 2015	Caries increment (dmft/dmfs), plaque scores
Pine et al. (2000)	Scotland	Primary schools	Randomised controlled trial	5‑year‑old children	461 children	Supervised school‑day brushing (1,000 ppm F) for 2 years; school & home incentive scheme; 6‑monthly dental exams; parental questionnaires	Standard practice (control)	24 months; published 2000	Caries increment; twice‑daily brushing frequency; parent attitudes and beliefs
Woodall et al. (2013)	England	Primary schools (18 schools)	Mixed‑methods service evaluation	Children aged 3–5; school staff; programme leads	18 schools (child numbers not consistently reported)	School‑day toothbrushing scheme; brushes/paste/storage ‘brush bus’; staff training; hygiene/consent protocols	None	Ongoing programme; report 2013	Acceptability; engagement; perceived behaviour change; process indicators

### BCTs in STPs

Across all included studies a consistent range of BCTs underpinned supervised toothbrushing. Table 2 summarises key BCTs in included studies mapped against the COM-B model of behaviour change.

**Table 2:** Supervised toothbrushing programmes: overview of BCTs of included studies.

Study	Behavioural background	BCTTv1	COM-B
Anishchuk and Waldron^17^	Explicitly designed with COM-B model of behaviour change Explicitly reported BCTs	1.1 Goal setting 1.4 Action planning 4.1 Instruction on how to perform the behaviour 5.1 Information about health consequences 6.1 Demonstration of the behaviour 7.1 Prompts/cues 8.1 Behavioural practice/rehearsal 8.3 Habit formation 9.1 Credible source 12.1 Restructuring physical environment 12.2 Restructuring social environment 12.5 Adding objects to the environment	Capability Opportunity Motivation
Petersen et al.^18^	Not explicitly reported BCTs	3.2 Social support (practical) 4.1 Instruction on how to perform the behaviour 5.1 Information about health consequences 7.1 Prompts/cues 8.1 Behavioural practice/rehearsal 9.1 Credible source 12.5 Adding objects to the environment	Capability Opportunity Motivation
Pine et al.^19^	Health Belief Model informed parts of the study Not explicitly reported BCTs	3.2 Social support 4.1 Instruction on how to perform the behaviour 5.1 Information about health consequences 7.1 Prompts/cues 8.1 Behavioural practice/rehearsal 10.1 Material incentive (behaviour) 10.4 Social reward 12.5 Adding objects to the environment	Capability Opportunity Motivation
Woodall et al.^20^	Not explicitly reported BCTs	3.2 Social support (practical) 4.1 Instruction on how to perform behaviour 7.1 Prompts/cues 12.5 Adding objects 12.1 Restructuring the physical environment	Capability Opportunity

Only one study, Anishchuk and Waldron^17^, was explicitly designed using the COM-B model of behaviour change. This particular study also identified and implemented specific BCTs and reported this information in their manuscript. Its resources included a storybook (“Brush Bunny”), toothbrushing song, poster, colourful “toothbrush bus” holders, guideline and summary cards, cross-infection standards, online training for dental hygienists and in-setting training for pre-school teachers, with a parental information card and a toothbrushing chart to support home routines.

The study by Petersen et al.^18^ in schools in Thailand included closely supervised after-lunch toothbrushing at school with every child supplied with a soft brush and 1,450 ppm fluoride toothpaste with 1.5% arginine and they were given additional supplies for home use. They also included two capacity-building workshops for teachers who then delivered classroom oral-health education, there was also regular communication and printed materials for parents (posters, music CDs, flipchart, games; bimonthly leaflets with monitoring questions); and periodic school monitoring visits.

In Pine et al.^19^, the Health Belief Model was utilised to guide parts of the intervention especially with regards to evaluating changes in parental attitudes and beliefs on oral health. Pine et al.^19^ included a supervised after-lunch toothbrushing intervention at school with short training for local “brushing supervisors”, a home incentive scheme with toothbrush/toothpaste, novelty holders, a toothbrushing chart with morning/bedtime boxes and stars, returning toothbrushing charts for rewards, certificates for being a “good toothbrusher”, a parent letter and incorporation of a cartoon character (“Sharkey”) on charts/certificates to remind children to continue toothbrushing.

Finally, in Woodall et al.^20^, their study of school-based supervised toothbrushing in 18 schools evaluated daily school-based supervised brushing in 3–5-year-olds with resources provided and replenished regularly (brushes, fluoride toothpaste, “brush bus” storage) alongside training for school staff following a flexible timing (not at start of day) and strong ongoing support from the oral-health team.

### Supporting home brushing through STP

None of the four included studies highlighted that BCTs can be incorporated into STP to influence at-home brushing behaviours. Despite their overall aims highlighting potential links with home brushing behaviour, from the four included studies, 3 specifically deployed surveys to look at home brushing and/or at home beliefs and attitudes regarding oral health. For example, Woodall et al.^20^ in their evaluation specifically focused on ‘whether the Toothbrushing in Schools scheme influences behaviour change in relation to toothbrushing within the home’. In Anishchuk and Waldron^17^ researchers specifically focused on home brushing behaviours and Pine et al.^19^ included parent questionnaires on beliefs and attitudes around oral health with incorporation of home brushing-related behaviours. For Petersen et al.^18^ the exploration of at home brushing was only through parent-feedback surveys.

In Anishchuk and Waldron^17^, in follow-up feedback surveys, 90% of parents the programme had helped their children brush better at home. Parents in general reported their children being more enthusiastic, spending longer toothbrushing and sharing the information with their siblings. A small number (6%) referred to the ‘Brush Bunny’ song saying their children sang along to it, used it to time their toothbrushing and it helped them concentrate on the toothbrushing.

In Pine et al.^19^, in feedback surveys, parents reported using the toothbrushing charts provided in the study during holidays stating that they were a good reminder for toothbrushing twice a day and that children enjoyed using them. Additional, positive feedback reported in this study concerned perceived benefits of toothbrushing charts, novelty toothbrushes and small gifts given to the children for regular toothbrushing. In Petersen et al.^18^ some parents reported that the printed material they received, especially a poster about toothbrushing for children, was particularly helpful to ensure that children brushed optimally. Finally, in Woodall et al. parents reported toothbrushing at home became easier as a result of the scheme because children were more aware of the importance of regular toothbrushing. Qualitative data from the same study highlighted a “ripple effect” whereby children passed on the information gained in school to their siblings and parents. This was also reported by Anishchuk and Waldron^17^ suggesting children transfer the information gained from school to their home and families.

From the overview of BCTs presented in Table 2, a range of specific techniques that can support home toothbrushing behaviour were identified within the four included studies. Techniques such as instruction and demonstration, repeated practice, and the use of cues build skills and habits in children and were mirrored at home. Table 3 presents a mapping exercise of identified BCTs that can support home toothbrushing delivered through STPs with practical examples provided within the four included studies. An additional mapping exercise identified relevant COM-B areas and TDF domains that encompass these BCTs to allow for greater transferability of examples in future projects.

**Table 3:** COM-B, TDF and BCT (BCTT v1) matrix offering practical examples for supporting Supervised Toothbrushing Programmes (STPs).

COM-B	TDF domain(s)	Recommended BCTs (BCTTv1 code)	How to apply in STPs (examples based on included studies)
Psychological Capability	Knowledge; Skills	Instruction on how to perform the behaviour (4.1); Demonstration (6.1); Behavioural practice/rehearsal (8.1); Credible source (9.1)	Brief, role-specific staff training with live demonstrations and practice based on a Standard Operating Procedure (SOP) pack.
	Memory, attention & decision processes; Behavioural regulation	Action planning (1.4); Problem solving (1.2); Review behaviour goals (1.5)	Co-create a class “toothbrushing plan” (who/when/where), anticipate and identify potential barriers, and check-in weekly.
Physical Opportunity	Environmental context & resources	Restructuring the physical environment (12.1); Adding objects to the environment (12.5)	Install labelled storage racks, closed storage, sink/flow layout (for ‘wet’ toothbrushing); provide toothbrushes and at least 1000 ppm fluoride toothpaste on site.
	Environmental context & resources	Prompts/cues (7.1)	Set a fixed daily slot with a song and visual timetable cards to prompt toothbrushing.
	Environmental context & resources (systems)	Restructuring the social environment (12.2); Action planning (1.4)	Formalise commissioning, supply chains and SOP adoption across settings (toolkit + rollout plan).
Social Opportunity	Social influences; Social/professional role & identity	Social support (practical) (3.2); Social comparison (6.2); Identification of self as role model (13.1)	Use “class champions” for peer-supervised toothbrushing and showcase classes with strong routines.
	Social influences; Environmental context & resources	Credible source (9.1); Framing/reframing (13.2)	Head-teacher endorsement frames STPs as part of the school’s health mission rather than “extra” work.
Reflective Motivation	Beliefs about consequences; Goals; Social/professional role & identity	Information about health consequences (5.1); Goal setting (behaviour) (1.1); Commitment (1.9); Framing/reframing (13.2)	Agree a whole-school goal (e.g., “Daily toothbrushing in all early years foundation stage classes”) and share simple “why this matters” briefs.
Automatic Motivation	Behavioural regulation; Emotion; Reinforcement	Habit formation (8.3); Prompts/cues (7.1); Social reward (10.4); Material incentive (10.1)	Embed the same daily routine, add recognition/rewards (class charts/stickers) to keep momentum.

## Discussion

This rapid review examined how STPs in early years and primary school settings utilise BCTs to promote oral health behaviours, and whether these school-based approaches influence children’s toothbrushing habits at home. A total of four studies were included. which described a diverse range of BCTs used to establish and reinforce toothbrushing behaviours, yet evidence directly linking school-based toothbrushing to improved home toothbrushing routines was limited. Despite this, qualitative findings suggested that STPs can indirectly enhance home practices through mechanisms such as modelling, habit formation, and social reinforcement.

The findings of this review reaffirm the well-established role of STPs in improving oral health outcomes among children. Included studies demonstrated that structured, daily supervised toothbrushing at school increases toothbrushing frequency, reduces caries experience, and improves attitudes towards oral health. The review extends these findings by mapping the active components of such programmes through the lens of behavioural science. When examined using the COM-B model and the Behaviour Change Technique Taxonomy (BCTTv1; 15), common intervention components included instruction, demonstration, practice, prompts and cues, and environmental restructuring—all targeting capability and opportunity domains. These components appear to underpin much of the success of STPs and highlight that behaviour change in oral health requires structured environmental and social support, not only knowledge provision.

A notable contribution of this rapid review lies in its systematic synthesis of how STPs may influence children’s behaviour beyond the school environment. While previous reviews have explored implementation barriers and facilitators^12,13^, few have explicitly examined the potential for school-based interventions to foster sustained toothbrushing at home. The evidence identified here suggests a “spillover” effect, where positive toothbrushing routines and knowledge acquired in schools may transfer to family settings. Parents reported greater child enthusiasm, increased toothbrushing duration, and use of cues learned at school—such as songs or charts—during home routines^17–20^. These findings align with social learning theory^21^, which posits that children adopt behaviours modelled and reinforced within social contexts, and with the notion of “behavioural contagion” within families. The role of teachers and peers as credible sources and the embedding of toothbrushing in daily routines appear especially important in translating learned behaviours from the classroom to the home.

Despite these promising indications, the review also highlights significant gaps in the evidence base. None of the included studies employed robust, quantitative measures of home toothbrushing behaviour, and none articulated theoretical mechanisms explaining how change in school settings might be generalised to the home. In most cases, parent-reported outcomes were anecdotal^18,19^. Moreover, while many interventions included take-home materials, parental letters, or incentive charts, the specific BCTs embedded in these tools were rarely documented or evaluated for fidelity. This absence of theoretically grounded process evaluation limits our understanding of the mechanisms of action responsible for behaviour change at home.

Future research should prioritise rigorous, theory-driven investigations into the link between supervised toothbrushing at school and sustained toothbrushing practices at home. Longitudinal mixed-methods studies employing validated behavioural measures could provide much-needed evidence on whether and how STPs influence at home brushing behaviour.

### Strengths and limitations

The key strength of this review was its rapid nature which allowed a quick yet robust review of available evidence to be conducted exploring the relationship between STPs and at-home toothbrushing behaviour. An additional strength was the utilisation of evidence-based tools like the BCTv1 taxonomy, COM-B and TDF that allow for transparency and provide steps for future research in this area. Limitations of this review lay within its rapid nature which in some cases, can limit the depth and breadth of data analysis, especially the lack of quality assessment of the available literature. Moreover, incorporation of more readily available databases should be considered in future reviews to ensure that all available research is accurately captured.

## Conclusion

In summary, this rapid review highlights that while supervised toothbrushing programmes effectively instil positive toothbrushing behaviours in nursery and school settings, the evidence for sustained change at home remains weak and poorly theorised. Nevertheless, the identification of key BCTs across successful programmes provides a practical foundation for designing future interventions that bridge school and home environments. Embedding behavioural science throughout the lifecycle of STPs—from conception and delivery to evaluation—offers a promising route to ensure that the benefits observed in educational settings are translated into long-term improvements in children’s oral health at home and beyond.

## Supplementary information

Appendix A

Appendix B

Appendix C

Appendix D

## Data Availability

The data supporting this article can be made available by the corresponding author upon request. A brief protocol prepared for the rapid review can be accessed upon request. This rapid review of evidence was not registered in any publicly available database.
