# Supplementary material for: Supervised toothbrushing programmes and at home brushing behaviour: a rapid review of evidence
Source: Evid Based Dent. 2026 Apr 10;27(2):42. doi: 10.1038/s41432-026-01218-y (PMC13309275; doi:10.1038/s41432-026-01218-y)
Supplement: Supplementary file 1 — Appendix A [file 41432_2026_1218_MOESM1_ESM.docx]

Appendix A

**MEDLINE via Ovid**

| 1 | (Child* or toddler* or early years).ti,ab. |
| --- | --- |
| 2 | (preschool* or nursery* or school* or primary school* or early childhood setting* or daycare* kindergarten*).ti,ab. |
| 3 | (supervised toothbrushing or toothbrushing program* or toothbrushing scheme* or toothbrushing club* or toothbrushing intervention* or toothbrushing club*).ti,ab. |
| 4 | 1 or 2 |
| 5 | 3 and 4 (143) |
| 6 | from 5 keep 1-4,6-9,12,14,16,19,22-25,30,32-33,36,38-42,44,46-47,49,54-55,57,59-60,62-63,67,69,73,75,79-82,84,88,92,94,97-98,100,102,104,106-107,118-119,121,123,128,134-135 |
| 7 | (parent* engag* or parent* involve or parent* particip* or home).ti,ab. |
| 8 | (link* or connection*).ti,ab. |
| 9 | 7 or 8 |
| 10 | 6 and 9 |

**Scopus**

(TITLE-ABS-KEY ( ( parent* engag* or parent* involve or parent* particip* or home ) )) AND (( TITLE-ABS-KEY ( ( supervised toothbrushing or toothbrushing program* ) ) OR TITLE-ABS-KEY ( ( toothbrushing scheme* or toothbrushing club* ) ) OR TITLE-ABS-KEY ( ( toothbrushing intervention* or toothbrushing club* ) ) ))

**PubMed**

(((preschool*[Title/Abstract] OR nursery*[Title/Abstract] OR school*[Title/Abstract] OR primary school*[Title/Abstract] OR early childhood setting*[Title/Abstract] OR daycare* kindergarten*[Title/Abstract]) OR (Child*[Title/Abstract] OR toddler*[Title/Abstract] OR early years[Title/Abstract])) AND (supervised toothbrushing[Title/Abstract] OR toothbrushing program*[Title/Abstract] OR toothbrushing scheme*[Title/Abstract] OR toothbrushing club*[Title/Abstract] OR toothbrushing intervention*[Title/Abstract] OR toothbrushing club*[Title/Abstract])) AND (parent* engag*[Title/Abstract] OR parent* involve[Title/Abstract] OR parent* particip*[Title/Abstract] OR home[Title/Abstract])

**APA PsycInfo** <1806 to July 2025 Week 1>
Search Strategy:

1     (Child* or toddler* or early years).ti,ab.
2     (preschool* or nursery* or school* or primary school* or early childhood setting* or daycare*
kindergarten*).ti,ab.
3     (supervised toothbrushing or toothbrushing program* or toothbrushing scheme* or toothbrushing club* or
toothbrushing intervention* or toothbrushing club*).ti,ab.
4     1 or 2
5     3 and 4
