# Supplementary material for: Supervised toothbrushing programmes and at home brushing behaviour: a rapid review of evidence
Source: Evid Based Dent. 2026 Apr 10;27(2):42. doi: 10.1038/s41432-026-01218-y (PMC13309275; doi:10.1038/s41432-026-01218-y)
Supplement: Supplementary file 2 — Appendix B [file 41432_2026_1218_MOESM2_ESM.docx]

Appendix B

Flow diagram

187 records identified by database searching

153 non-duplicate records titles and abstracts screened

16 eligible publications full text screened

4 publications included in the rapid review

34 duplicates removed

137 excluded due to failure to meet inclusion criteria

12 excluded as they did not mention link to home brushing
