# Supplementary material for: Supervised toothbrushing programmes and at home brushing behaviour: a rapid review of evidence
Source: Evid Based Dent. 2026 Apr 10;27(2):42. doi: 10.1038/s41432-026-01218-y (PMC13309275; doi:10.1038/s41432-026-01218-y)
Supplement: Supplementary file 4 — Appendix D [file 41432_2026_1218_MOESM4_ESM.docx]

Appendix D.

Addressing the updated recommendations for the Cochrane rapid review methods guidance for rapid reviews of effectiveness

| Recommendations | Response |
| --- | --- |
| **Topic refinement: Setting the research question** |  |
| 1 Involve knowledge users to set and refine the review question, eligibility criteria, and outcomes of interest, with consultation at various stages of the review | Experts guided the selection of the rapid review question |
| 2 Develop a protocol that includes the review questions, population, interventions, comparators, outcomes, and methods of conducting the review | A working protocol was developed at the start of the rapid review process |
| **Topic refinement: Setting the eligibility criteria** |  |
| 3 Clearly define the eligibility criteria, including any restrictions or limits: |  |
| 3.1 Limit the number of interventions and comparators | Information can be found on page 4 |
| 3.2 Limit the number of outcomes, focusing on those most important for decision making | Information can be found on pages 4-5 |
| 3.3 Consider restriction of the search date of the evidence base, with clinical or methodological justification provided | Information can be found on page 4 |
| 3.4 Limit the setting, with clinical or methodological justification provided | Information can be found on pages 4-5 |
| 3.5 Limit the publication language to English at study selection, with other languages added when relevant | Information can be found on page 4 |
| 3.6 Prioritise the inclusion of high quality study designs relevant to the review question or objective | Information can be found on pages 4-5 |
| **Searches** |  |
| 4 Involve an information specialist to develop the search strategy and to consider search methods, resources, and search limits | Research team included experts within their fields and researchers in experience (ZM, KG-B, SE-Y) with experience in rapid reviews |
| 5 Select a small number (but at least two) bibliographic databases that are likely to retrieve relevant literature For rapid reviews focused on randomised controlled trials only:   - Use a combination of two of the following databases (if you have access): Medline, CENTRAL, and Embase3967-69   Additional considerations:   - For Cochrane rapid reviews of health interventions, where search strategies are always designed by an information specialist and peer reviewed using the PRESS statement 37 (see recommendation 6), using CENTRAL as the primary database is recommended. This is a highly concentrated source of reports of randomised controlled trials and quasi-randomised controlled trials. In many regions, CENTRAL is free through the Cochrane Library.40 In addition, it is accessible to Cochrane members through the Cochrane Register of Studies Online (<https://crso.cochrane.org/>).41 Additional searches of Medline (eg, through PubMed) and possibly Embase (if access is available) may be limited to the previous two months to capture the most recently published studies as CENTRAL is currently only updated once a month93942 - If CENTRAL and Embase are not available, consider a search of Medline combined with an appropriate supplementary search (eg, a study register such as ClinicalTrials.gov, using the PubMed similar articles feature), although this might not be appropriate for all topics914 | Information can be found on pages 4-5 and appendix A |
| For other rapid reviews that include non-randomised studies:   - Database selection should be carefully considered for rapid reviews depending on available time and resources. In many cases, Medline will be the most relevant database, but this is not always the case.1043 A search of specialised databases (eg, CINAHL, PsycInfo, ERIC) may be necessary for specialised review topics (eg, the use of CINAHL for rapid reviews related to nursing care, PsycInfo for rapid reviews related to mental health, or ERIC for rapid reviews related to educational interventions) | Information can be found on pages 4-5 and appendix A |
| 6 Use the PRESS checklist to peer review the primary search strategy If use of PRESS is not possible, at a minimum search strategies should be double checked for typographical errors, missed key words, and overall structure | PRESS checklist was not used however, all searches were checked by 2 reviewers with a 3^rd^ independent reviewer available to resolve any issues |
| 7 Assess the need for grey literature and supplemental searching. Justify the sources to be searched | Need for grey literature was assessed as not important for this particular topic at this particular time |
| **Study selection** |  |
| Screening of title and abstract and of full text 8 Employ piloting exercises at abstract and full text screening levels to allow team members to test the study selection process on a selective sample of records to ensure that all team members apply a consistent approach to screening | Information can be found on pages 4-5 |
| 9 Conduct dual and independent screening of a proportion of records (eg, 20%) and assess reviewer agreement—if agreement is good (eg, κ is ≥0.8), proceed with single screening | Information can be found on page 4 |
| **Data extraction** |  |
| 10 Limit data extraction to only the most important data fields relevant to address the review question | Information can be found on pages 4-5 |
| 11 For data extraction, employ a piloting exercise to allow team members to test this task on a small proportion of records to ensure that all team members perform it consistently and correctly | Information can be found on page 5 |
| 12 Have one person extract the data, and for critical data that can affect the results or conclusions, have a second person verify the data for accuracy and completeness | We utilised two reviewers for all screening and data extraction with a 3^rd^ reviewer available to resolve any disagreements |
| 13 When available, extract data directly from existing systematic reviews rather than from primary studies | N/A |
| **Risk of bias assessment** |  |
| 14 Use validated and study design specific tools to assess the risk of bias of included studies | N/A as discussed, no risk of bias appraisal was undertaken for this work |
| 15 Focus the risk of bias assessment at least on the most important outcomes | N/A |
| 16 Have one person perform the risk of bias assessment and a second person to verify the judgements | N/A |
| **Synthesis** |  |
| 17 Provide a descriptive summary of the included studies | Information can be found on table 1 |
| 18 Provide a synthesis of the findings | Information can be found throughout ‘results’ section |
| 19 Consider a meta-analysis if appropriate and resources permit | N/A |
| 20 Consider how to synthesise evidence when including one systematic review or more | N/A |
| **Certainty of evidence** |  |
| 21 Use the GRADE approach to assess the certainty of evidence if time and resources allow | N/A |
| 22 Limit the certain of evidence ratings to the main intervention and comparator, and focus on critical outcomes only | N/A |
| 23 Have one person complete the GRADE assessment and a second person to verify assessments | N/A |
| **Other best practice considerations** |  |
| 24 Provide a clear description of the selected review approach, which includes outlining the restricted methods used. Additionally, discuss the potential limitations of these chosen methods and how they may influence the interpretation of the research findings | Information can be found under ‘limitations’ |
